# Comparing the Efficacy of 3% NaCl With Pressure Dressing and Oral Glycopyrrolate With Pressure Dressing in the Treatment of Parotid Sialocele in Postoperative Oral Squamous Cell Carcinoma Patients: Study Protocol of a Randomized Controlled Trial

**DOI:** 10.7759/cureus.65237

**Published:** 2024-07-24

**Authors:** Jayesh Phalak, Deepankar Shukla, Nitin Bhola

**Affiliations:** 1 Department of Oral and Maxillofacial Surgery, Sharad Pawar Dental College and Hospital, Datta Meghe Institute of Higher Education and Research, Wardha, IND

**Keywords:** reconstruction, head and neck, antisialogogue, management, parotid sialocele

## Abstract

Introduction

Parotid sialocele is a common complication following surgery for oral squamous cell carcinoma (OSCC), leading to discomfort and potential complications. Various treatment modalities have been proposed, including 3% NaCl with pressure dressing and oral glycopyrrolate with pressure dressing, yet evidence comparing their efficacy is limited. This randomized controlled trial aims to compare the efficacy of 3% NaCl with pressure dressing and oral glycopyrrolate with pressure dressing in the treatment of parotid sialocele among postoperative OSCC patients undergoing neck dissection.

Material and methods

Patients diagnosed with OSCC undergoing surgical intervention resulting in parotid sialocele will be randomly assigned to receive either 3% NaCl with pressure dressing or oral glycopyrrolate with pressure dressing. Treatment efficacy will be assessed based on the discharge of any serous collection from the suture line, the length of time required for resolution of salivary sialocele, wound dehiscence, the length of the postoperative stay, the amount of time to resume oral feeds, and the timing of postoperative adjuvant therapy.

Results

This is an ongoing study protocol, and we are expecting the analysis of the results in 2026.

Conclusion

This randomized controlled trial will furnish invaluable insights into the comparative efficacy of 3% NaCl with pressure dressing and oral glycopyrrolate with pressure dressing in the treatment of parotid sialocele among postoperative OSCC patients. The findings will inform evidence-based decision-making and optimize treatment strategies for this common complication.

## Introduction

Saliva is an integral part of oral health. It keeps the environment hypotonic, which aids in taste perception, and controls the oral cavity's pH and ionic makeup, which are essential for salivary proteins to function properly. In addition to breaking down carbohydrates by cleaving 1,4-glycosidic bonds, salivary amylase has the ability to bind streptococci and facilitate their removal from the mouth. In addition, other proteins such as lactoferrin, lysozyme, peroxidase, and secretory IgA help prevent tooth caries, candidiasis, and periodontal disease [1]. Managing postoperative salivary leaks is a major problem for maxillofacial surgeons. With any ablative surgery, there is a chance of salivary leakage where there is a discontinuity in the mucosal or capsular salivary barrier following the oropharyngeal and hypopharyngeal malignancies, complete parotidectomy, total laryngectomy, and lesion involving resection of part of the oral cavity [2].

During a neck dissection, numerous surgeons opt to incorporate the parotid tail within the upper extent of the dissection. The sectioned portion of the parotid gland may pose a risk for sialocele formation and subsequent salivary fistulas following the neck dissection. Prolonged hospital stays lead to salivary leaks, psychological impact on the patient, delays in adjuvant care and oral feeding, a higher incidence of surgical infection and free flap failure, and higher treatment expenses [3]. After surgical reconstructions of the head and neck using local or regional flaps, studies have found that between 13.5% and 29% of patients experience salivary fistulas [4-7]. Numerous treatment methodologies, both aggressive and conservative, have been proposed with differing levels of morbidity and success. Management options encompass the utilization of pressure dressings and antisialagogues, radiation therapy, tympanic neurectomy, total parotidectomy, intraoral transposition of the parotid duct, administration of botulinum toxin A, and application of fibrin glue and other sclerosing agents [8-12].

This randomized controlled trial (RCT) aims to compare the efficacy of 3% NaCl with pressure dressing and oral glycopyrrolate with pressure dressing in the treatment of parotid sialocele among postoperative oral squamous cell carcinoma (OSCC) patients undergoing neck dissection.

## Materials and methods

Study design

The present study will be a prospective RCT conducted on patients requiring treatment of parotid sialocele at the inpatient and outpatient departments of the Oral and Maxillofacial Surgery Department at Sharad Pawar Dental College and Hospital (SPDC), DMIHER, Sawangi (Meghe), Wardha, India, from June 2024 to May 2026. The study will be conducted in accordance with the Helsinki Declaration. Additionally, the study received clearance from the Institutional Ethical Committee of Datta Meghe Institute of Medical Sciences on February 11, 2024, with ethical approval number DMIMS(DU)/IEC/2024/228. The trial was registered with the Clinical Trials Registry of India (CTRI) with registration number CTRI/2024/06/068599.

Participants

A total of 50 patients who have been operated on for OSCC and are undergoing neck dissection will be screened, and 24 patients diagnosed with parotid sialocele postoperatively will be included in the study. The patients enrolled in the study must fulfill the following criteria, shown in Table 1.

**Table 1:** Inclusion criteria

Inclusion Criteria
Patients treated for oral squamous cell carcinoma undergoing neck dissection (buccal and GB sulcus) with resection of the parotid tail followed by reconstruction using a suitable flap
Patients having fluctuant swelling over the preauricular
Patients with excessive salivation postoperatively in patients operated for oral squamous cell carcinoma will be included in the study

Patients who cannot participate in the study are shown in Table 2.

**Table 2:** Exclusion criteria

Exclusion Criteria
Patients who have systemic or autoimmune diseases affecting the salivary gland
k/c/o hypertension
Salivary gland neoplasms or inflammatory disorders
Previously operated for oral squamous cell carcinoma
Recurrence cases of oral squamous cell carcinoma
Traumatic injuries to the salivary gland region
Patients not willing to give consent for the study will be excluded from the study

The preoperative evaluation includes obtaining written informed consent, recording clinical history, and conducting a thorough clinical examination. Routine blood investigations (hemoglobin, clotting time, bleeding time, random blood sugar (RBS)) will be performed. Patients would be explained regarding the study protocol following the preoperative evaluation.

Randomization

This will be a prospective interventional study. This is a randomized, active-controlled trial with two parallel groups, A and B, who will be allocated by randomization using a computer-generated table. Group A will comprise patients in whom parotid sialocele treatment will be done with 3% NaCl with pressure dressing. Group B will comprise patients in whom parotid sialocele treatment will be done with oral glycopyrrolate with pressure dressing.

Intervention

The clinical study will be performed at the Department of Oral and Maxillofacial Surgery, Siddharth Gupta Memorial Cancer Hospital, Sawangi, Wardha, Maharashtra, India, after obtaining approval from the IEC, DMIHER (DU) and explaining detailed treatment protocol to the patients. The procedure will be performed in patients who have been histopathologically confirmed to have OSCC. A total of 24 patients fitting into the criteria of the study will undergo the procedure of assessment and surgery. History will be taken and the pre-treatment clinical condition of the patient will be assessed based on the clinical, radiological, and histopathological data. Records will be maintained for the evaluation of parameters. All the patients are ready to be taken for surgery after the presurgical workup, which includes pre-anesthesia checkup (PAC) fitness, thoroughly explained written and signed consents, patient preparation, pre-op photographs, and induction.

The subjects would be divided randomly into two groups. Group A would include 12 subjects clinically diagnosed with parotid sialocele operated for OSCC undergoing neck dissection; they will undergo injection with 3% NaCl in the intra-parotid region postoperatively. Group B would include 12 subjects clinically diagnosed with parotid sialocele treated for OSCC undergoing neck dissection and will undergo oral/through RT glycopyrrolate therapy postoperatively. A solution of 3% hypertonic saline will be meticulously prepared and subsequently subjected to heating within an autoclave, attaining a temperature of 60 °C. The fundamental rationale for employing hypertonic saline at this precise temperature lies in its capacity to instigate the denaturation process within the underlying gland. This denaturation, in turn, leads to a discernible reduction in saliva secretion, thereby facilitating the desired blockage of the gland. In the postoperative period, after confirmatory diagnosis of parotid sialocele, 60 °C hypertonic saline of 5 mL will be injected, and immediately, barrel pressure dressing will be applied. Injection will be given every day until the drain obtained will be 24 mL/day. For Group B, postoperatively on confirmatory diagnosis of parotid sialocele, 6 mg/day of glycopyrrolate will be given orally/through RT for the reduction of the saliva secretions and thus blocking the gland. Intermediate barrel pressure dressing will be applied. The reduction of salivary secretion will be observed at intervals of 3, 5, 7, and 15 days, with a postoperative follow-up period of up to a month.

Outcome measures

Outcome variables such as postoperative sialocele formation, discharge from the suture line, drain collection, the length of postoperative hospital stay, wound healing, duration required to resumption of oral feeds, and timing of postoperative adjuvant therapy will be evaluated, as shown in Table 3.

**Table 3:** Outcome measures

Outcome Measures
Postoperative sialocele formation will be diagnosed clinically by the primary surgeon through physical examination of the index region on days 1, 3, 5, and 7 postoperatively, with follow-up extended up to one month.
Discharge from the suture line will be monitored daily, and the amount will be measured by soaking gauge pads.
Drain collection will be measured in milliliters from the day of surgery until drainage ceases, and the postoperative day of drain removal will be recorded.
The length of the postoperative hospital stay will be calculated from the day of surgery to patient discharge.
Wound healing will be assessed using the REEDA scale on days 1, 3, 5, and 7 postoperatively, with follow-up continuing for one month.
The duration until oral feeds are resumed and the timing of postoperative adjuvant therapy will also be documented.

Any postponement exceeding six weeks in a patient's postoperative radiation therapy due to wound healing issues would be considered a disruption or delay in adjuvant postoperative therapy. Early salivary sialoceles referred to occurrences within 30 days of the primary procedure, whereas late instances were those arising more than 30 days after the primary procedure.

Sample size

The minimum sample size required for piloting the study, as no previous studies were available for power analysis, will be 12 samples per group for the comparative evaluation of the results between the groups [13].

Statistical analysis plan

All analyses will utilize RStudio software version 4.3.2 (Posit, Boston, Massachusetts). The complete dataset will include participants who meet the study's inclusion and exclusion criteria, ensuring no missing data across all parameters. The analysis will focus on the following outcome variables such as postoperative sialocele formation, discharge from the suture line, drain collection, length of postoperative hospital stay, wound healing, time to resumption of oral feeds, and timing of postoperative adjuvant therapy.

Descriptive statistics will include the mean, standard deviation (SD), minimum, maximum, standard error (SE), and 95% confidence intervals (CI) for parametric data. The Kolmogorov-Smirnov test will be employed at a 5% significance level to assess normality. Parametric tests will be applied if data are normally distributed; nonparametric tests will be used otherwise. For non-normally distributed data, summary measures will include the median as well as the lower and upper quartiles.

For normally distributed continuous variables, the significance between pre- and post-intervention groups will be evaluated using t-tests (α = 0.05). Non-normally distributed continuous variables will be analyzed using the Mann-Whitney U test to determine significance. Frequency (N) and percentages (%) will summarize categorical variables, and significance will be assessed using the chi-square test at a 5% significance level (α = 0.05).

The results will be presented with appropriate statistical metrics and p-values to indicate significance levels. Tables and figures will be utilized for a clear and transparent data presentation.

## Results

This RCT is poised to make a substantial contribution to the current body of knowledge concerning the management of parotid sialocele in patients who have undergone surgery for OSCC. This is an ongoing study protocol, and the results are going to be collected and analyzed in early 2026.

## Discussion

The primary goal of this study is to ascertain if oral glycopyrrolate or 3% NaCl, the two drugs primarily employed as therapy options for parotid sialocele, are superior to each other in any manner. A study conducted by Bhattarai et al. demonstrated that due to its cost-effectiveness, hypertonic saline is a successful option for fistula closure due to its ready availability, non-toxicity, non-irritating nature to surrounding tissues, lack of foreign body or hypersensitivity reactions, and minimal risk of damaging the facial nerve and its branches [14].

In a study conducted by Chhabra et al. in 2009, they explored the utilization of warm, hypertonic saline injections for the closure of fistulas. There is a distinct advantage to this method in that it sidesteps potential complications such as foreign body reactions or hypersensitivity in patients, as it does not involve the use of chemical substances. Additionally, it is readily available, cost-effective, and does not pose toxicity or irritation risks to surrounding tissues. Furthermore, there is a minimal risk that it can injure the facial nerve, and it promotes gland parenchyma fibrosis, leading to the spontaneous closure of the fistula without complications. However, the authors emphasized that only two cases were treated with hypertonic saline for parotid fistulae at their institution, and more extensive clinical experience with a larger patient cohort is necessary before definitive recommendations can be made [15].

Aisha et al. (2015) concluded that utilizing a blend of compression barrel dressing and hot hypertonic saline injection emerges as a cost-effective and highly successful strategy for managing parotid sialocele. This treatment is favorably received by patients and demonstrates a notable absence of significant complications [16].

A study conducted by Medexus Pharmaceuticals, Inc. and reviewed by the Canadian Agency for Drugs and Technologies in Health in July 2020 examined the efficacy of glycopyrrolate oral solution (Cuvposa) for the treatment of chronic severe drooling in pediatric patients with neurological conditions. Glycopyrrolate is a synthetic anticholinergic agent classified as a quaternary ammonium compound that has limited ability to cross the blood-brain barrier. It works by blocking the effects of acetylcholine on the salivary glands, thereby decreasing saliva production. As a competitive inhibitor, glycopyrrolate targets muscarinic receptors in the salivary glands, reducing salivation by preventing the activation of these receptors [17].

## Conclusions

This RCT aims to evaluate the comparative efficacy of 3% NaCl with pressure dressing versus oral glycopyrrolate with pressure dressing for managing parotid sialocele in postoperative OSCC patients. This study addresses a critical gap in clinical knowledge regarding optimal treatment strategies for this challenging complication.

By employing a rigorous methodology, including randomization, we strive to provide robust evidence on which intervention offers superior outcomes in terms of sialocele resolution, patient comfort, and overall treatment effectiveness. The findings from this trial are expected to inform clinical practice by guiding healthcare providers in selecting the most effective therapeutic approach for managing parotid sialocele postoperative OSCC surgery, potentially improving patient outcomes and enhancing the quality of care in this patient population.
